# Arbuscular Mycorhizal Fungi Associated with the Olive Crop across the Andalusian Landscape: Factors Driving Community Differentiation

**DOI:** 10.1371/journal.pone.0096397

**Published:** 2014-05-05

**Authors:** Miguel Montes-Borrego, Madis Metsis, Blanca B. Landa

**Affiliations:** 1 Department of Crop Protection, Institute for Sustainable Agriculture (IAS-CSIC), Cordoba, Spain; 2 Tallinn University, Institute of Mathematics and Natural Sciences, Tallinn, Estonia; Institute for Plant Protection (IPP), CNR, Italy

## Abstract

**Background:**

In the last years, many olive plantations in southern Spain have been mediated by the use of self-rooted planting stocks, which have incorporated commercial AMF during the nursery period to facilitate their establishment. However, this was practised without enough knowledge on the effect of cropping practices and environment on the biodiversity of AMF in olive orchards in Spain.

**Methodology/Principal Findings:**

Two culture-independent molecular methods were used to study the AMF communities associated with olive in a wide-region analysis in southern Spain including 96 olive locations. The use of T-RFLP and pyrosequencing analysis of rDNA sequences provided the first evidence of an effect of agronomic and climatic characteristics, and soil physicochemical properties on AMF community composition associated with olive. Thus, the factors most strongly associated to AMF distribution varied according to the technique but included among the studied agronomic characteristics the cultivar genotype and age of plantation and the irrigation regimen but not the orchard management system or presence of a cover crop to prevent soil erosion. Soil physicochemical properties and climatic characteristics most strongly associated to the AMF community composition included pH, textural components and nutrient contents of soil, and average evapotranspiration, rainfall and minimum temperature of the sampled locations. Pyrosequencing analysis revealed 33 AMF OTUs belonging to five families, with *Archaeospora* spp., *Diversispora* spp. and *Paraglomus* spp., being first records in olive. Interestingly, two of the most frequent OTUs included a diverse group of Claroideoglomeraceae and Glomeraceae sequences, not assigned to any known AMF species commonly used as inoculants in olive during nursery propagation.

**Conclusions/Significance:**

Our data suggests that AMF can exert higher host specificity in olive than previously thought, which may have important implications for redirecting the olive nursery process in the future as well as to take into consideration the specific soils and environments where the mycorrhized olive trees will be established.

## Introduction

Spain is the world's largest olive oil producer, accounting for more than one-third of global production [1], [2]. In Andalusia (Southern Spain), olive orchards dominate the landscape in an impressive monoculture that covers approximately 17% of the total surface of the region (1.5 million ha) [1], [3]. In this region, different olive farming systems can be found including: i) *conventional farming* with rain fed orchards of low plant density, intensive tillage, and low inputs in fertilizer, as well as intensive drip-irrigated orchards, grown with higher inputs of pesticides and fertilizers in order to push up olive yields, and ii) *organic farming* using no chemical inputs and mainly non or light-tillage and use of a vegetative cover to prevent soil erosion [3], [4], [5]. Additionally, in the last years, many new olive plantations have been mediated by the use of self-rooted planting stocks which have incorporated commercial arbuscular mycorrhizal fungi (AMF) in the potting mixture during the nursery period to facilitate establishment [6], [7], [8] due to their beneficial effects against biotic and abiotic stresses [9], [10], [11], [12], [13].

One of the critical steps for applying AMF to improve crop health is the appropriate selection of effective and well adapted-isolates to be used as inoculants. Although it is well known that olive tree is a mycotrophic plant [7] there is not enough knowledge concerning the effect of cropping practices, olive genotype and environment (soil type and climate) on the biodiversity of AMF in olive orchards in the Mediterranean Region. Knowledge of those factors may be essential to take advantage on the use of AMF in modern oliviculture.

In the present study we have examined the structure and diversity of AMF communities in the rhizosphere of cultivated and wild olives in Andalusia, southern Spain, by using two culture-independent molecular approaches: Fluorescent terminal restriction fragment length polymorphism (T-RFLP) analyses of amplified 28S rDNA sequences and SSU rDNA amplicon parallel 454 pyrosequencing. We also have determined which agronomic or environmental factors associated to the olive orchards sampled are the main drivers of the AMF structure.

## Materials and Methods

### Ethics Statement

No specific permits were required for the described field studies. Location of organic olive orchards was provided by the Andalusian Committee of Organic Farming (CAAE, Junta de Andalucía). Permission to sample the olive orchards were granted by the landowner. The samples from wild or feral forms of olive are located in public areas or degraded formations and abandoned groves. The specific location of all samples from the study is provided in Table S1. The 96 olive orchards sampled in this study were also included in previous studies [5], [14] in which the bacterial communities and functional diversity of the olive rhizosphere was assessed. The sites are not protected in any way. The areas studied do not involve any species endangered or protected in Spain.

### Location of Olive Orchards and Rhizosphere Sampling

Soil and roots samples were collected from 90 commercial orchards differing in management system [conventional (49 orchards) vs. organic (41 orchards)] located in the main olive-growing areas of Córdoba (41 orchards), Granada (3 orchards), Jaén (34 orchards), and Sevilla (12 orchards) provinces in Andalusia, southern Spain [5; Table S1]. In addition, six samples (LO, LOBA, BAETICA, MACO, LOMCO, EPCO) from three sites each in Córdoba and Cádiz provinces containing wild or feral forms of olive ‘Acebuches’ (i.e., secondary sexual derivatives of the cultivated clones or products of hybridization between cultivated trees and nearby oleasters) were included in the study [14; Table S1]. Orchards across all locations sampled differ in climate, soil texture and physicochemical characteristics, soil management system (use of cover crops vs. bare soil), and irrigation regimen (rain-fed vs. drip-irrigated). When possible, we tried to sample a representative distribution of the above considered factors within each orchard management system. Detailed description of soil physicochemical properties, and agronomic and climatic characteristics of the sampled orchards is provided in Table S1. Some of the soil physicochemical and climatic characteristics of the sampled locations were provided recently [5], [14].

Root (only young and active) and soil samples were collected in May to July 2009 as described by Aranda et al. [14] and Montes-Borrego et al. [5] in the area of the canopy projection from the upper 5 to 30 cm of soil from three different points around each individual tree. Eight trees per orchard were sampled, and all roots from all trees were thoroughly mixed to obtain a single representative sample per orchard. Intact root systems were shaken gently by hand to remove all but the soil close- and naturally-adhering to the plant root and were kept at 5°C until processing.

Additionally the geographic location and altitude of the sampling sites were determined using a global positioning system (GPS), and climatic variables of each sampling site were obtained from SigMapa, Geographic Information System from the Spanish Ministry of “Medio Ambiente y Medio Rural y Marino” (http://sig.mapa.es/geoportal/) using ArcGIS 10 (ESRI, Redlands, California, EE.UU.) (Table S1).

### DNA Extraction from Rhizosphere Samples

Pooled olive root samples were cut into 1-cm pieces with a sterile scissors to get a uniform sample per location. Rhizosphere suspensions (including rhizosphere soil and rhizoplane) were obtained by vigorously shaking 2 g of root segments (four independent replications) suspended in 20 ml of sterile distilled water in an orbital shaker for 10 min and sonicated (Ultrasons, JP Selecta SA, Barcelona, Spain) for 10 minutes. Then, 3 ml of those rhizosphere suspensions were subjected to two consecutive centrifugations at 11,000 rpm for 4 min and the pellet was kept at −20°C until processed. DNA from each of the four rhizosphere soil pellets (approximately 200 mg; four replication per each of the 96 olive orchards) was extracted using the PowerSoil DNA Isolation Kit (MO BIO Laboratories, Inc., Carlsbad, USA) and the FastPrep-24 (MP Biomedicals, Inc., Illkirch, France) instrument run at 6.0 m/s for 40 s as described elsewhere [14].

### T-RFLP Analysis

For T-RFLP analysis PCR amplification of partial LSU of rDNA from mycorrhiza were performed using a nested-PCR approach, the first PCR round employing 20 ng of template and the primer pair LR1/FLR2 [15] and the second one the primer pair FLR3/FLR4 [16] following conditions described by Mummey and Rillig [17]. Primer FLR3 was 5′ end-labeled with the fluorescent dye FAM. T-RFLP analysis was performed for all samples using 5 µl of PCR products (about 1000 ng) and *Taq*I restriction enzyme (Fast Digest, Fermentas, Germany) in a final volume of 10 µl. *Taq*I restriction enzyme was selected from those (*Alu*I, *Mbo*I and *Taq*I) that were shown to discriminate more AMF groups in a previous study [17] after preliminary testing with a subset of our rhizosphere samples (*data not shown*). Terminal restriction fragments (TRF) were loaded and separated on a 3130XL genetic analyzer (Applied Biosystems, California, USA) at the SCAI-University of Córdoba sequencing facilities. Size of fragments were determined using a ROX500 size standard, and matrices containing incidence as well as peak area data of individual TRFs were generated for all samples with GeneMapper software (Applied Biosystems). Peaks of less than 100 fluorescence units (FU) and shorter than 50 bp were not included in the analysis to eliminate primer dimmers and other small charged molecules. Similarly, molecules that were not present in at least two of the four replicate profiles were disregarded. Also, TRFs that differed by less than 1 bp were clustered, unless individual peaks were detected in a reproducible manner. TRFs profiles were standardized based on methods described previously by Dunbar et al. [18]. The relative abundance of each TRF was calculated as the ratio of the peak area for that TRF to the sum of peak areas for all TRFs in the profile and was expressed as a percentage. Diversity statistics were calculated from standardized profiles of rhizosphere samples by using the number and area of peaks in each profile as representative of the number and relative abundance of OTUs, as defined by Dunbar et al. [19]. Phylotype richness was calculated as the total number of distinct TRF sizes (with length between 50 and 500 bp) in a profile and the Shannon-Wiener diversity index was calculated as described before [14]. Finally, a single standardized T-RFLP profile for each orchard was produced by averaging peak area for each TRF from four replicates.

### Pyrosequencing Analysis

For 454-pyrosequencing SSU rRNA Glomeromycota sequences were amplified from a DNA mixture obtained from the four independent rhizosphere DNA extractions per olive orchard. This approach was taken to ideally cover as much biodiversity as possible and to ensure that representative AMF communities from each olive location were sampled [20]. The pyrosequencing was performed as described in Davison et al. [21] using a two-step PCR protocol with the primers NS31 and AML2, which target a ca. 560-bp central fragment of the SSU rRNA gene in Glomeromycota [22], the most widely used marker in AMF surveys to date [23], [24]. These primers were linked to partial sequencing primers A and B, respectively. Bar-code sequences, 8 bp in length, were inserted between the A primer and NS31 primer sequences. Thus, the composite forward primer was: 5′-GTCTCCGACTCAG(NNNNNNNN) *TTGGAGGGCAAGTCTGGTGCC*-3′; and the reverse primer was 5′-TTGGCAGTCTCAG *GAACCCAAACACTTTGGTTTCC*-3, where partial sequences of A and B primers are underlined, barcode is indicated by N-s in parentheses and specific primers NS31 and AML2 are shown in italic. Then, a 10x dilution of the first PCR product was used in a second PCR where full sequencing adapters were added (Primer A 5′-CCATCTCATCCCTGCGTGTCTCCGAC-3′ and Primer B 5′-CCTATCCCCTGTGTGCCTTGGCAGT -3′). The reactions contained 5 µl of Smart-Taq Hot Red 2x PCR Mix (Tartu, Naxo Ltd, Estonia), 1 µl of extracted DNA, and 0.2 µM of each primer in a final volume of 10 µl. The reactions were performed using a Thermal cycler 2720 (Applied Biosystems) under the following conditions: 95°C for 15 min; five cycles of 42°C for 30 s, 72°C for 90 s, 92°C for 45 s; 35 (first PCR) or 20 (second PCR) cycles of 65°C for 30 s, 72°C for 90 s, 92°C for 45 s; followed by 65°C for 30 s and 72°C for 10 min. PCR products were separated by electrophoresis using 1.5% agarose gels in 0.5 x TBE, and the PCR products were purified from the gel using the Qiagen QIAquick Gel Extraction kit (Qiagen Gmbh, Germany) and further purified with AgencourtH AMPureH XP PCR purification system (Agencourt Bioscience Co., Beverly, MA, USA). The 96 quantified samples were finally mixed at equimolar concentrations prior to sequencing. GATC Biotech (Constanz, Germany) performed sequencing procedures as custom service using a Genome Sequencer FLX System and Titanium Series reagents (Roche Applied Science, Indianapolis, IN, USA). Sequencing of 96 samples was performed as a part of a bigger dataset.

### Processing of Pyrosequencing Data and phylogenetic analysis

Pyrosequencing data were processed as described by Fierer et al. [25] using the Quantitative Insights Into Microbial Ecology (QIIME) toolkit [26]. In brief, fungal sequences were quality trimmed, assigned to rhizosphere samples based on their barcodes and denoised using default parameters. Chimeras were identified with uclust_ref software [27] and removed, and the remaining sequences were binned into OTUs using a 97% identity threshold with uclust_ref software. Then, to take into account the different number of sequences obtained for each orchard sample in the pyrosequencing analysis we estimated the relative frequency of each OTU in each orchard. Next, the most abundant sequence from each OTU was selected as a representative sequence for that OTU and deposited in the Genbank database under accessions numbers KF831296-KF831328 and the entire dataset of reads in the Sequence Read Archive of Genbank under BioProject ID PRJNA237741. Alpha diversity statistics including Richness (numbers of OTUs) and the Shannon index were also determined for orchard samples with at least five sequences.

Taxonomy was assigned to OTUs by using the Basic Local Alignment Search Tool (BLAST) for each representative sequence against the Silva 108 database (http://www.arb-silva.de/documentation/release-108/) as well as by BLAST search against the Maarj*AM* database (http://maarjam.botany.ut.ee/, [23]). Sequences from the representative set of AMF OTUs obtained in this study, the reference AMF database from Redecker and Raab [28], the blast hits from Silva 108 and the Maarj*AM* databases, and those AMF sequences reported in olive from Calvente et al [7] and present in the GenBank database were aligned using ClustalW software [29] with default parameters. Sequence alignments were manually edited using BioEdit software [30]. Phylogenetic analysis of the sequence data sets was performed based on maximum likelihood (ML) and Bayesian inference (BI) using MrBayes version 3.1.2 software [31]. The best fitted model of DNA evolution was obtained using jModelTest v. 2.1.1 [32] with the Akaike information criterion (AIC). The Akaike-supported model, the base frequency, the proportion of invariable sites, and the gamma distribution shape parameters and substitution rates in the AIC were then used in phylogenetic analyses. BI analysis under a general time reversible of invariable sites and a gamma-shaped distribution (TIM2 +I+G) model for the SSU rRNA, were run with four chains for 1.0×10^6^ generations.

### Statistical Analysis

The rank-based Kruskall-Wallis test was used to determine differences in the Richness and Shannon diversity indexes in relation to the different agronomic factors of the olive orchard evaluated using the Statistical Analysis System software package (SAS version 9.2; SAS Institute, Cary, NC, USA). Non-metric multidimensional scaling (NMDS) analyses were performed using MetaMDS functions within the vegan package of R software (R Core Development Team, 2005) [33] based on dissimilarities calculated using the Bray–Curtis index obtained for T-RFLP and pyrosequencing results, using 1,000 runs with random starting configurations, and environmental variables (agronomic and climatic characteristics and soil physicochemical properties) were fitted using the envfit routine. For data derived from pyrosequencing analysis only the Glomeromycota sequences were used. Ordinations for the Bray–Curtis dissimilarity derived from relative frequency of OTUs in the pyrosequencing analysis did not reach acceptable [34] stress and stability levels and was not performed. Instead, a Multivariate Regression Tree (MRT) was calculated. MRT are a statistical technique that can be used to explore, describe, and predict relationships between multispecies data and environmental characteristics. MRT forms clusters of sites by repeated splitting of the data, with each split defined by a simple rule based on environmental variables. The splits are chosen to minimize the dissimilarity of sites within clusters [35]. The sums of squares multivariate regression tree was calculated within the mvpart package with the R software, using the one-standard error rule on the cross-validated relative error to determine the number of terminal nodes [35].

## Results and Discussion

### Diversity of olive AMF communities

**Figure 1:**
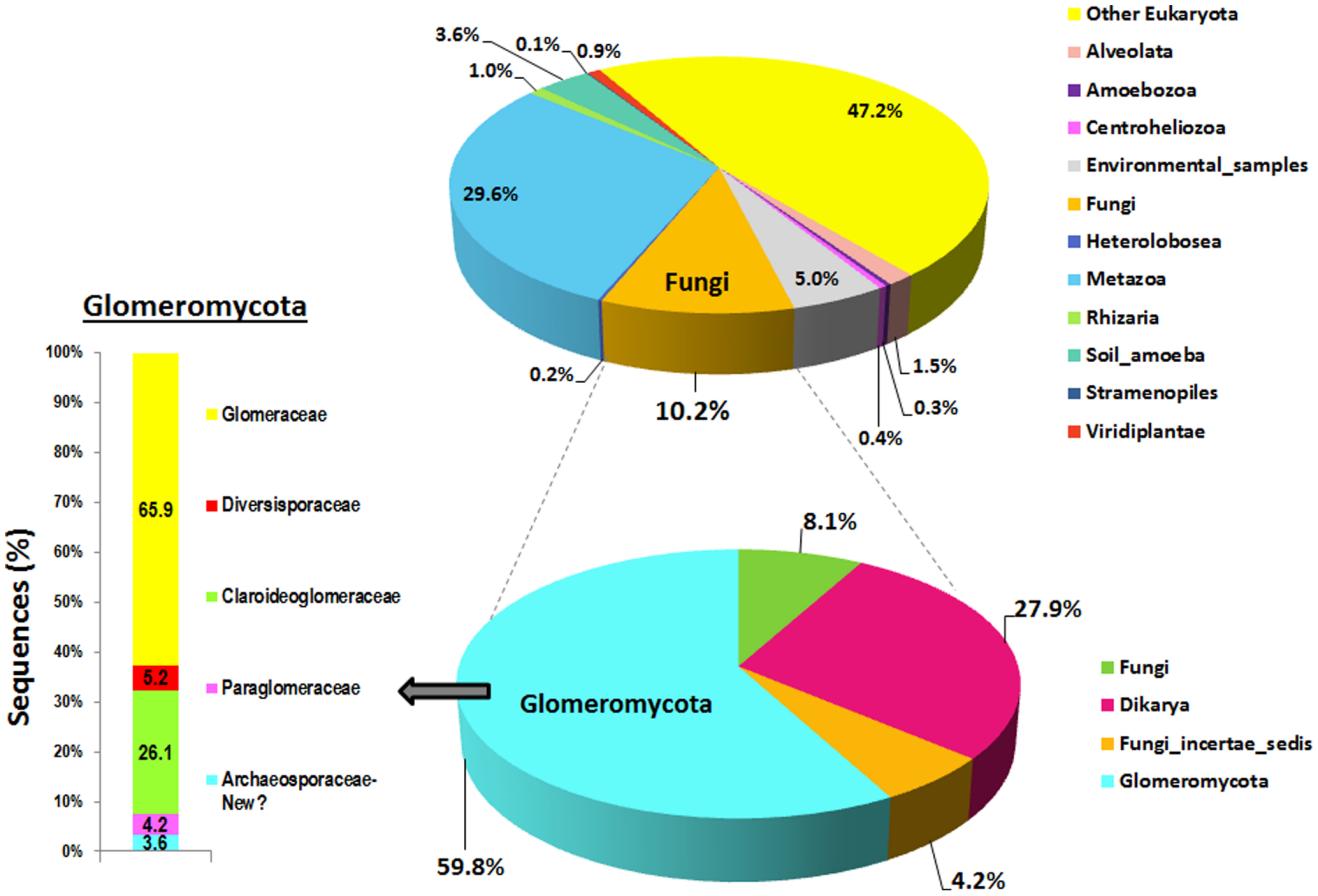
Proportion of overall phyla and disectioning of the fungal and Glomeromycota phyla detected by pyrosequencing analysis with primers NS31/AML2 from rhizosphere samples obtained from 96 olive orchards in Andalusia, southern Spain.

- **T-RFLP analysis.** A total of 36 unique TRFs profiles were consistently identified in the 384 rhizosphere samples analyzed by T-RFLP analysis, with 30 TRFs (83.3%) found in a reproducible manner in 93 of the 96 olive orchards sampled. Mean Richness values ranged from 1 to 15 depending of the rhizosphere sample with an average of 5 TRFs per olive orchard. This translates into an estimated total of 30 different OTUs present across all sampled orchards with 13 and 3 OTUs being common in at least 25% or 50% of olive orchards, respectively. Mean Shannon diversity index values ranged from 0.3 to 2.2 (Figure S1; Table S2). We did not find significant differences (*P*>0.120) in AMF richness or Shannon diversity indexes derived from T-RFLP analysis according to the orchard management system, use of irrigation, presence of vegetative cover, olive tree variety or olive age (Figure S1). It has been shown that although diversity indexes are useful in describing community characteristics, they do not provide information of important compositional features of biodiversity relating to the abundances of shared taxa [36] and statistical analyses that incorporate taxon abundance and identity are more appropriate to specifically assess changes in microbial community composition and to identify the existence, if any, of agronomic or environmental gradients.
- **Pyrosequencing analysis.** The pyrosequencing approach of the 96 composite samples yielded a total of 13,772 high-quality reads after denoising, with a length >100 bp and <550 bp, a mean of 147 sequences per orchard field, and two samples with no sequences (Table S2). Of these, most of sequences 47.2% could not be assigned to any Eukaryota Phyla, 29.6% were assigned to the Phylum Metazoa, whereas 10.2% (1,108 reads) could be assigned to OTUs from fungal families (Figure 1), which indicates that primers NS31 and AML2 are not enough specific for amplifying AMF sequences. Other studies using pyrosequencing analysis have also shown that in spite of the supposed AMF primer specificity, ‘contaminant’ sequences belonging to taxa different from *Glomeromycota* are detected. For example, Öpik et al. [37] using same NS31 and AML2 primers and Ballestrini et al. [38] and Lumini et al. [20] using NS31 and AMmix primers also found about >55% of amplified sequences belonging to taxa from non-*Glomeromycota* fungi. In a recent work Kohout et al. [39] also demonstrated that a combination of up to five primer sets specifically designed to amplify *Glomeromycota,* including primer NS31/AML2, co-amplifies to a high extend non-target AMF sequences including plant, Asco- and Basidiomycota (ca. 20 to 50%, depending of the primer set used). In our study, due to the fact that we did not retrieve *Glomeromycota* sequences from some olive orchards our results could be somehow biased. Consequently, to improve the number of sequences and molecular species characterization of AMF, new designed specific AMF-primers should be tested in complex matrixes such as soil or rhizosphere [39], [40], or deeper sequencing effort should be done in future studies to face this problem and to capture the total AMF diversity in olive.

The fact that we extracted DNA from the olive rhizosphere (i.e., soil tightly adhered to roots) might have accounted for the low presence of AMF sequences; probably extracting DNA from washed or entire roots may enhance the specificity of AMF amplification. In our study 59.84% of sequences assigned to fungi belonged to *Glomeromycota* (Figure 1), with 38 olive orchard samples retrieving no *Glomeromycota* sequences (Table S2). We did not find any pattern for the lack of *Glomeromycota* sequences in those samples with any of the agronomic, climatic or soil physicochemical properties of the sampled orchards (*data not shown*). When defining an OTU as belonging to AMF on the basis of having at least 97% similarity to sequences classified as AMF in the Silva and GenBank databases we identified 33 OTUs that could be unequivocally assigned to the *Glomeromycota* in 58 out of 94 olive rhizosphere samples (with a mean of 11.4 sequences per orchard) (Figure 1; Table 1; Figure S2; Table S2). Mean richness values ranged from 1 to 9 with an average of 2.7 OTUs per olive orchard. Mean Shannon diversity index values ranged from 0 (1 single OTU) to 2.7 (Figures S1 and S2; Table S2). We did not find significant differences (*P*>0.230) in AMF richness or Shannon diversity indexes derived from pyrosequencing analysis according to the farm soil management system, use of irrigation, presence of vegetative cover, olive tree variety or olive age (Figure S1).

**Table 1:** Glomeromycota taxa detected in olive rhizosphere samples, taxonomic affiliation, number of sequences from each taxa, frequency of occurrence in the sampled olive orchards, and the closest related AMF sequence.

Family	OTU identification^a^			Number of sequences	Frequency of sequences (%)	Frequency of orchards (%)	Closest taxa^b^	
	Phylogenetic group	Code	Acc. Number				Silva 108	Maarj*AM*
Archaeosporaceae	Ia	OAMF127	KF831299	23	3.47	10.34	*Glomeromycota* sp.	*Archaeospora* sp. VT5
	Ib	OAMF64639	KF831323	1	0.15	1.72	*Archaeospora trappei*	*Archaeospora trappei* VT245
Paraglomeraceae	IIa	OAMF131	KF831300	1	4.07	1.72	*Fungi*	*Paraglomus laccatum* VT281
	IIb	OAMF60009	KF831322	27	0.15	6.90	*Fungi*	*Paraglomus brasilianum* VTX239
Claroideoglomeraceae	IIIa	OAMF26258	KF831310	52	7.84	18.97	*Glomus etunicatum*	*Glomus* sp. group B VT193
(*Glomus* group B)	IIIb	OAMF216	KF831306	121	18.25	27.59	*Glomus etunicatum*	*Glomus geosporum* VT65
		OAMF71	KF831324	1	0.15	1.72	*Glomus etunicatum*	*Glomus* sp. group B VT56
		OAMF333	KF831315	1	0.15	1.72	*Glomus etunicatum*	No match
		*Total*		123	18.55	27.59		
Diversisporaceae	IVa	OAMF79857	KF831327	19	2.87	3.45	*Glomus eburneum*	*Glomus* sp. MO-GC1 VT60
(*Glomus* group C)	IVb	OAMF264	KF831311	20	3.02	12.07	*Glomus eburneum*	*Glomus* sp. Wirsel OTU20 VT62
		OAMF156	KF831302	1	0.15	1.72	*Glomus eburneum*	*Diversispora* sp. VT62
		*Total*		21	3.31	13.79		
Glomeraceae	V	OAMF19034	KF831303	16	2.41	1.72	*Glomeromycota*	*Glomus* sp. Dictamnus2 VT163
(*Glomus* group A)		OAMF443	KF831318	11	1.66	5.17	Uncultured *Glomus* sp.	*Glomus* sp. Douhan6 VT143
		*Total*		27	4.07			
	VI	OAMF43	KF831317	23	3.47	5.17	Uncultured *Glomus* sp.	*Glomus* sp. VT145
	VIIa	OAMF359	KF831316	1	0.15	1.72	*Glomeromycota*	*Glomus* sp. VT153
		OAMF91246	KF831328	124	18.70	36.21	Uncultured *Glomus* sp.	*Glomus* sp. VT118
		*Total*		125	18.85	36.21		
	VIIb	OAMF73	KF831325	59	8.90	15.52	Uncultured *Glomus* sp.	*Glomus* Wirsel VT137
	VIIc	OAMF499	KF831320	9	1.36	1.72	Uncultured *Glomus* sp.	*Glomus* JP3 VT128
	VIIIa	OAMF213	KF831305	12	1.81	3.45	*Glomeromycota*	*Glomus* sp. Alguacil09b VT109
	VIIIb	OAMF452	KF831319	11	1.66	8.62	Uncultured *Glomus* sp.	*Glomus* sp. Glo45 VT109
	IXa	OAMF22696	KF831307	14	2.11	6.90	Uncultured *Glomus* sp.	*Glomus* sp. Glo24 VT105
		OAMF15	KF831301	2	0.30	3.45	*Rhizophagus intraradices*	*Glomus intraradices* VT113
		OAMF22729	KF831308	29	4.37	8.62	*Rhizophagus intraradices*	*Glomus intraradices* VT113
		*Total*		45	6.79	18.97		
	IXb	OAMF293	KF831312	8	1.21	6.90	Uncultured *Glomus* sp.	*Glomus* sp. VT94
	IXc	OAMF123	KF831297	2	0.30	3.45	Uncultured *Glomus* sp.	No match
	IXd	OAMF521	KF831321	17	2.56	8.62	Uncultured *Glomus* sp.	*Glomus* sp. Glo7 VT214
	Xa	OAMF102182	KF831296	4	0.60	1.72	*Glomus* sp.	*Funneliformis caledonium* VT65
	Xb	OAMF77556	KF831326	5	0.75	1.72	*Glomus* sp.	*Funneliformis mosseae* VT67
	Xc	OAMF311	KF831314	13	1.96	10.34	Uncultured *Glomus* sp.	*Septoglomus viscosum* VT63
	XI	OAMF3	KF831313	1	0.15	1.72	Uncultured *Glomus* sp.	*Glomus* sp. VT301
		OAMF20	KF831304	4	0.60	3.45	Uncultured *Glomus* sp.	*Glomus* sp. VT301
		*Total*		5	0.75	3.45		
	XII	OAMF25566	KF831309	30	4.52	18.97	*Glomeromycota*	*Glomus* Alguacil09c Glo4 VT166
		OAMF125	KF831298	1	0.15	1.72	*Glomeromycota*	*Glomus* Alguacil09c Glo4 VT166
		*Total*		31	4.68	18.97		

a The phylogenetic groups were arbitrarily named according to their position in the Bayesian analysis shown in Figure 2. Each AMF OTU sequence found in the study was assigned a Code (OAMF# S#) where OAMF refers to ‘olive arbuscular mycorrhizal fungi’ and # to the number assigned to each representative AMF OTUs derived from uclust_ref analysis with the QIIME software.

b Closest taxa assigned by BLAST analysis using the Silva 108 or Maarj*AM* data base. Numerical codes of ‘virtual taxa’ VT as appear in the Maarj*AM* database are shown as in Figure 2.

1. **Comparison of T-RFLP and pyrosequencing analysis.** Although in the pyrosequencing analysis we got some orchard samples with no *Glomeromycota* sequences and this technique is costly, labour-intensive and allows lower number of samples to be processed, it provides some advantages over the T-RFLP analysis. For example, the latter technique does not provide any information on the taxa identified, different taxa (species) may share similar TRFs in electropherograms, and multiple TRFs profiles can exist within a single species [41]. Furthermore, the lack of specififity of the primers used for T-RFLP have also been shown in other studies and may also be a source of errors when PCR products serve as basis for those fingerprinting approaches [39], [41]. Consequently, data dervived from T-RFLP analysis should be complemented with other techniques that provide taxa identity information such as library cloning or pyrosequencing as was the case of this study.

### Species identity of olive AMF communities

In our study the 33 OTUs identified represented most of the major AMF lineages, including *Paraglomus* spp. (family Paraglomeraceae; two OTUs, comprising 4.20% of reads and 6.90% of fields), *Glomus* group A (family Glomeraceae; 22 OTUs, comprising 59.73% of reads and 81% of fields), *Glomus* group B (family Claroideoglomaceae; four OTUs, 26.40% of reads and 38% of fields), *Glomus* group C (family Diversisporaceae; three OTUs, 6.03% of reads and 14% of fields), and *Archaeospora* (two OTUs, 3.62% and 12% of fields) (Figures 1 and 2; Table 1); with *Archaeospora* spp., *Diversispora* spp. and *Paraglomus* spp. being first records in olive. It should be noted that OTU OAMF127 clustered with the virtual taxon sequence AF131054 that has been recently proposed as a potential new taxon (new family or even order) within Glomeromycota [37].

**Figure 2:**
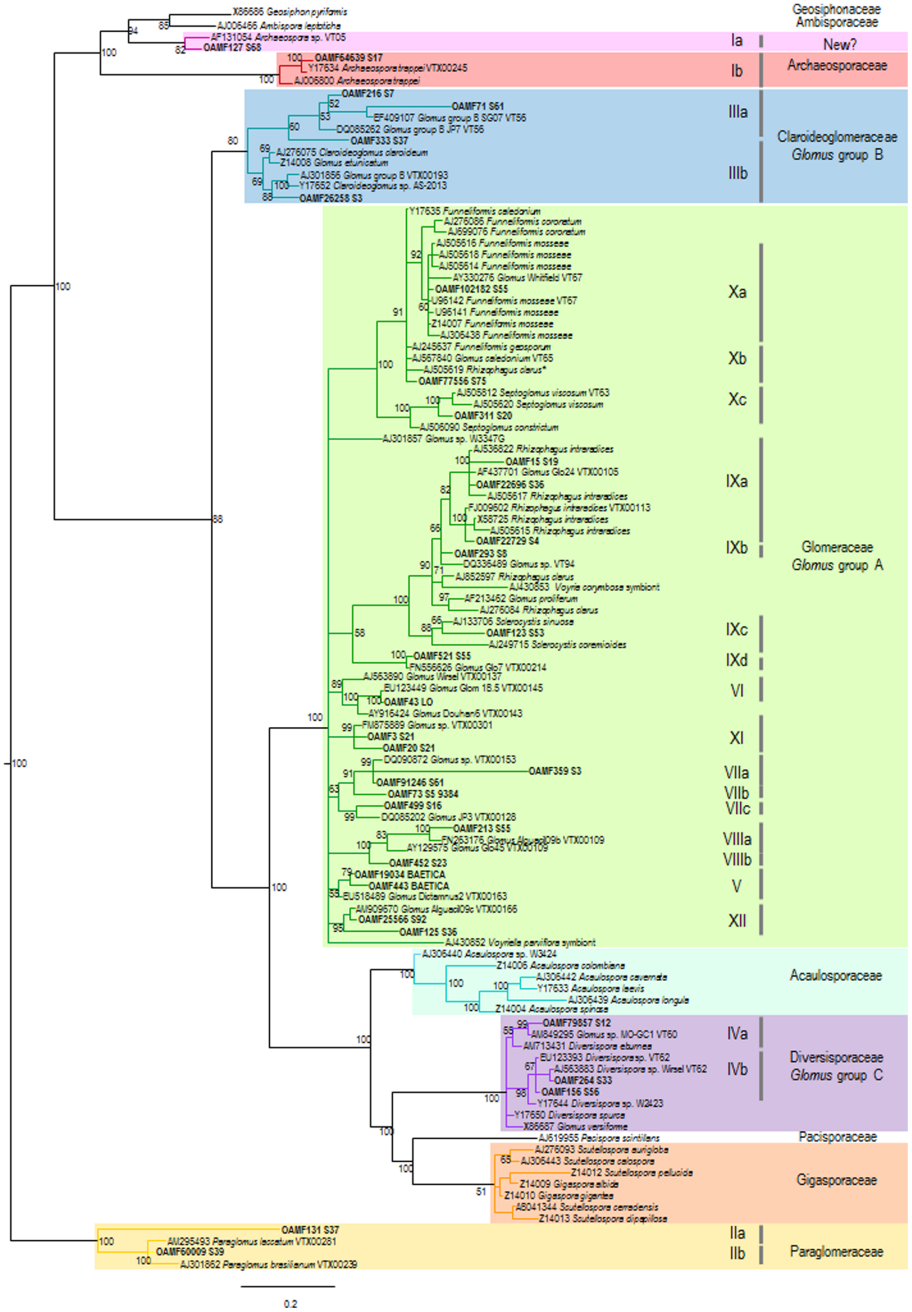
Phylogenetic relationships of nuclear small subunit ribosomal RNA (SSU rRNA) gene sequences of *Glomeromycota* reference sequences derived from uclust_ref search with those that matched the silva_108 and Maarj*AM* databases, the reference AMF database from Redecker and Raab [28] and those reported in olive from Calvente et al [7] and present in the GenBank. Bayesian 50% majority rule consensus tree as inferred from nSSU rRNA sequences alignments under the general time reversible + G + I model. Numbers on the nodes indicate Bayesian posterior probabilities (>50%). The phylogram was rooted with Paraglomeraceae sequences. Numerical codes in bold name each representative AMF OTUs from olive rhizosphere derived from uclust_ref analysis with the QIIME software and are labelled (OAMF# S#) where OAMF refers to ‘olive arbuscular mycorrhizal fungi’ and # to the number assigned and groups identified and the remaining code refers to the soil sample. Phylogenetic groups (I to Xd) were arbitrarily described and are shown in Table 1. (*) Although this sequence was originally identified as belonging to *R. clarus* by Calvente et al. [7] its closest taxonomic affiliation is to *Funneliformis* sp. and clearly differs from sequences AJ276084 and AJ852597 of *R. clarus*.

The fact that most sequences from our study belonged to the *Glomeraceae* family (*Glomus* group A) that contains several cryptic taxa with differences in ecological properties agrees with other studies that have found many isolates of this group in different locations through the world, including Mediterranean-type environments, on both natural woodlands to high input managed agro-ecosystems [20], [42], [43], [44] suggesting that these taxa have a generalist ruderal style with tolerance to disturbance such as in agricultural ecosystems.

Interestingly, we found that those AMF species commonly used as olive inoculants or previously isolated from olive roots (i.e., *Claroideglomus claroideum, Funneliformis mosseae, Rhizophagus clarus*, *R. intraradices,* and *Septoglomus viscosum*; see [7], [8], [9], [12], [13]) showed low abundance since they were present in 1.7 to 18.97% of fields. This is in agreement with the fact that AMF belonging to Glomeraceae family colonize preferentially the roots and might be present in lower densities in the rhizosphere soil [45]. On the contrary, two AMF sequences including OAMF216 belonging to Claroideoglomeraceae (18.6% of sequences), and OAMF91246, a new unidentified Glomeraceae (18.9% of sequences) that formed a separate cluster from other well-known AMF Glomeraceae taxa, were identified in 27.6% and 36.21% of olive orchards, respectively (Figure 2; Table 1). This might indicate that AMF can exert higher host specificity in olive than previously thought which may have implications for the olive nursery process. Thus, some authors have reported a differential growth response of olive cultivars to AMF inoculation where this responsiveness to mycorrhization has been found to depend on both the AMF species and the plant genotype [7], [46]. In our study, we did not find any clear differences between the AMF sequences detected in the rhizosphere of wild olives and those found in the cultivated ones (Figure S2). This could be due to the small number of sequences that we sampled from wild olives which deserves further studies since wild olives have been shown to be a potential reservoir for discovering microbial species of diverse biotechnological and commercial interest [14], [47].

### Factors shaping the structure of AMF communities in olive rhizosphere

**Figure 3:**
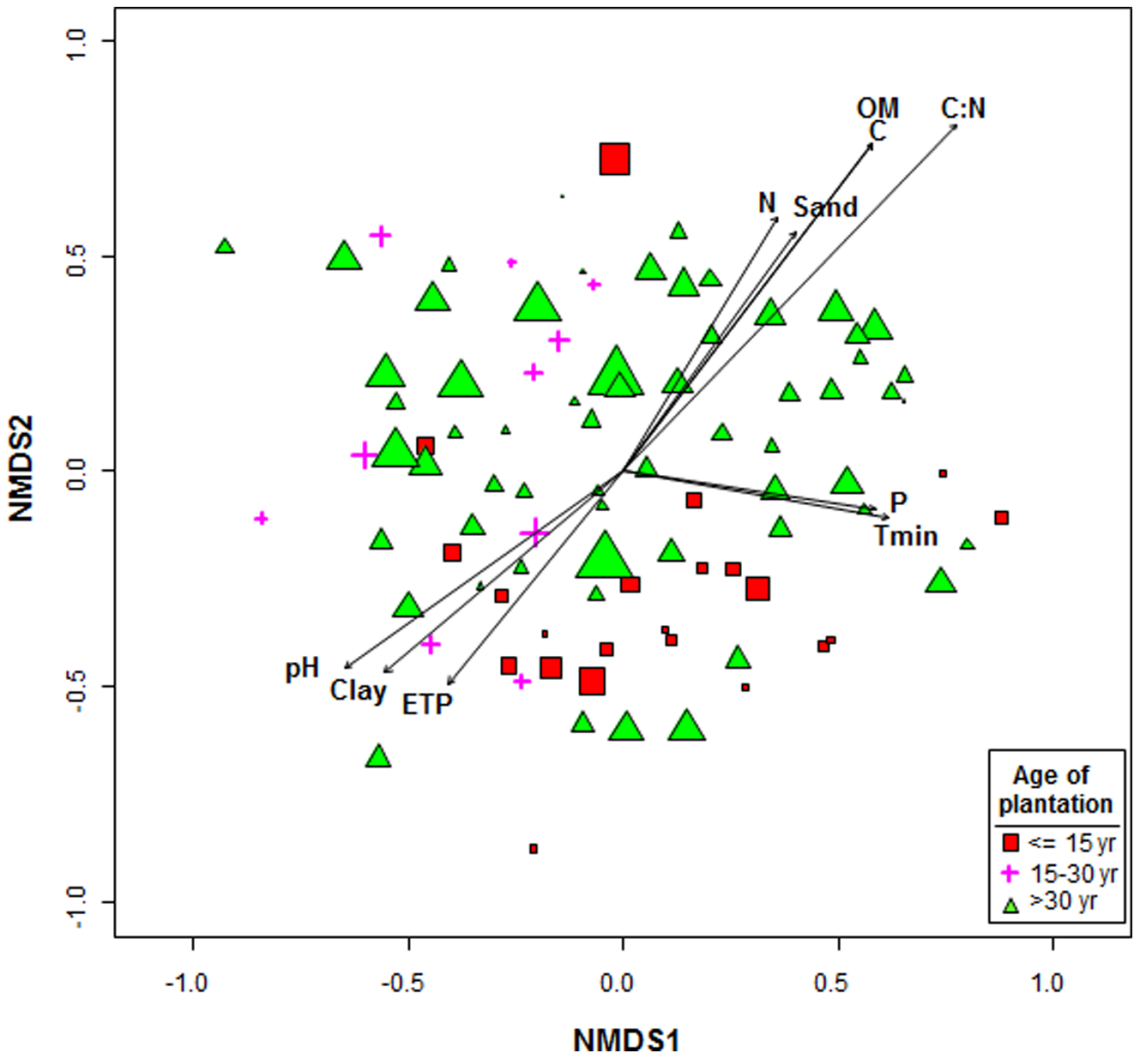
NMDS biplot of a Bray-Curtis dissimilarity matrix of T-RFLP analysis. The fitted vectors of environmental and physicochemical soil variables and the agronomic variable age of plantation (indicated with different symbols) most significantly and strongly associated (*P*<0.05) with the ordination and shown in Table 2 are also represented. Size of symbols is proportional to AMF richness in those olive orchards.

It has been shown that although diversity indexes (such as Richness and Shannon used in our study) are useful in describing community characteristics they do not provide information of important compositional features of microbial diversity related to the abundances of shared taxa [36] which migth explain that we did not find an effect of the environmental and agronomic variables on the estimated alpha-diversity indexes. Consequently, in a second approach to specifically assess changes in AMF community composition (incorporating taxon abundance (frequency) and identity), we used NMDS ordination to represent, in two dimensions, the pairwise Bray-Curtis dissimilarities between AMF communities derived from T-RFLP analyses. Then, we projected each of the environmental and agronomic variables independently onto the NMDS ordination to identify hypothetical gradients likely related to the differentiation in AMF composition (Figure 3; Table 2). In relation to agronomic variables AMF communities were differentiated according to the cultivar genotype and age of plantation and the irrigation regimen of the olive orchard, in that order, whereas the grouping according to the orchard management system or presence of a vegetative cover was not significant (Table 2). Thus, there was a tendency to locate rhizosphere samples in the NMDS ordination from olive orchards <15 year old at the bottom quandrant of Y = 0 (with only two exceptions), whereas olive orchards of 15 to 30 year old were all located on the left cuadrant of X = 0 (Figure 3). The effect of olive gentoype in affecting soil biota has also been shown in a recent study [48] which demostrated that olive genotypes significantly influenced the nematode assemblages present in their rhizospheric soil.

**Table 2:** Summary of relationships^a^ between agronomic, soil and environmental factors and AMF communities assessed by T-RFLP analysis.

Factors b	*r^2^*	*P*	
*Soil physicochemical properties*			
** Clay (%)**	**0.1087**	**0.003996**	**
** Sand (%)**	**0.0987**	**0.007992**	**
** Organic C (%)**	**0.1915**	**0.000999**	***
Organic N (%)	0.1006	0.010989	*
Extractable P (ppm)	0.0735	0.02997	*
Exchangeable K (ppm)	0.0315	0.228771	
CEC	0.0005	0.976024	
** C:N ratio**	**0.2623**	**0.000999**	***
** pH(KCl)**	**0.1301**	**0.000999**	***
** SOM (%)**	**0.1914**	**0.000999**	***
*Climatic characteristics*			
Total Rainfall	0.0183	0.438561	
Average Rainfall	0.0045	0.811189	
ETP	0.0849	0.026973	*
Tmax	0.0486	0.117882	
Tmin	0.0815	0.016983	*
Tmean	0.0412	0.154845	
Altitude	0.0274	0.306693	
*Agronomic characteristics*			
** Olive variety**	**0.2836**	**0.000999**	***
Presence of vegetative cover	0.0032	0.738262	
** Age of plantation**	**0.1233**	**0.001998**	**
Irrigation regimen	0.0401	0.033966	*
Orchard management system	0.002	0.986014	

a Correlations with all environmental variables (*r* ^2^) were obtained by fitting linear trends to the NMDS ordination obtained with each restriction enzyme and significance (*P*) was determined by permutation (nperm  = 1000).

‘***’ = *P*<0.001;

‘**’ = *P*<0.01;

‘*’ = *P*<0.05. Variables with highest significant weight are shown in bold.

b Orchard agronomic and climatic characteristics, and soil physicochemical properties are shown in Table S1 and some of them were reported before [5], [14]. Climatic variables were obtained from SigMapa, Geographic Information System from the Spanish Ministry of “Medio Ambiente y Medio Rural y Marino” (http://sig.mapa.es/geoportal/) using ArcGIS 10 (ESRI, Redlands, California, EE.UU.).

We also identified C:N ratio, soil C and organic matter content and pH as the environmental variables better explaining (*P*<0.001; 0.2836>*r^2^*>0.1301) the AMF community composition among the olive orchards, in that order (Figure 3, Table 2). Other environmental factors showing a significant (*P*<0.034) but lower effect included clay, sand and N content, extractable P and annual evapotranspitation and minimum temperature of the sampled locatios (Table 2).

**Figure 4:**
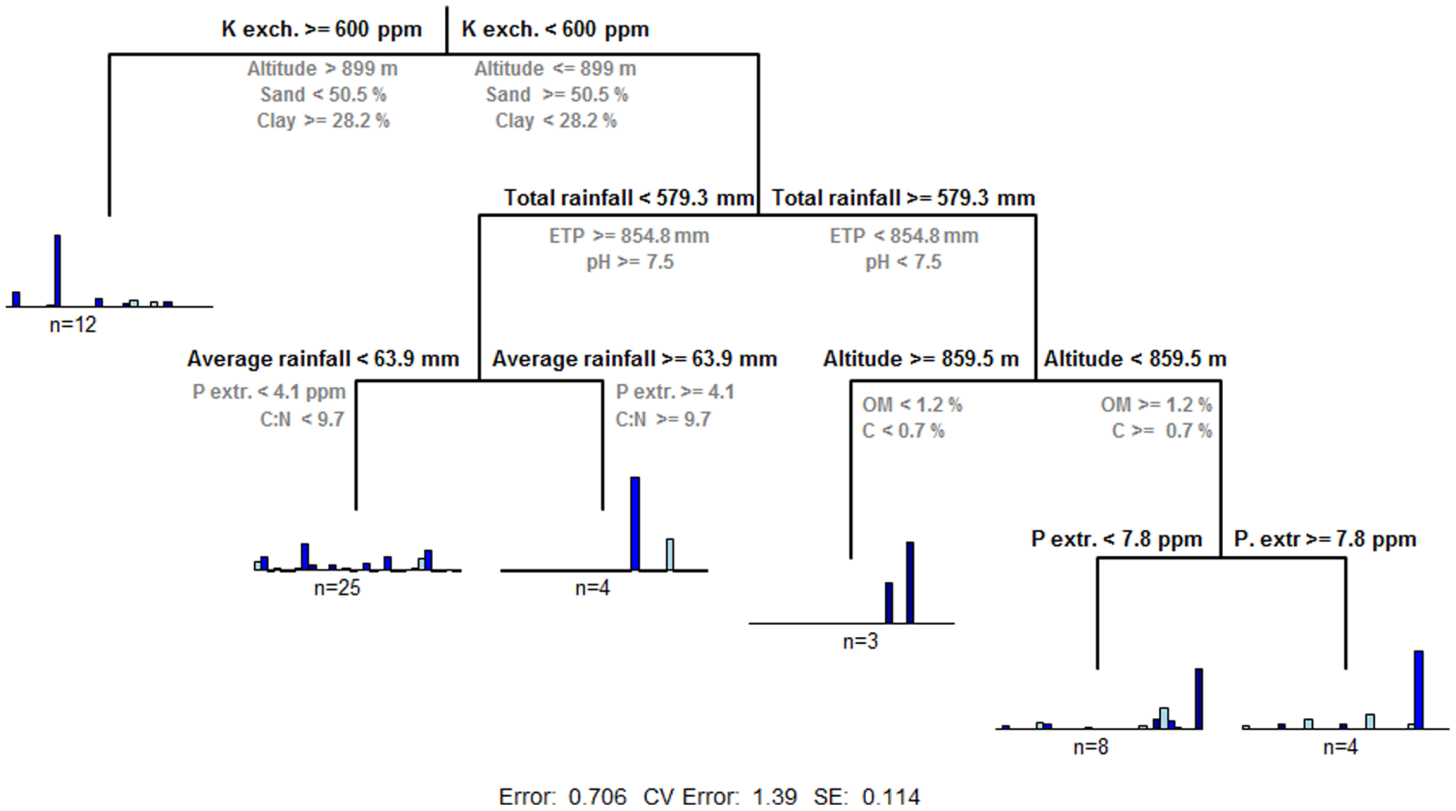
Sums of squares multivariate regression tree summarizing olive AMF community–agronomic, environmental and soil factors relationships. The tree was calculated using frequency of AMF OTUs derived from pyrosequencing analysis (). For each split a rule is selected based on the predictors to minimize the dissimilarity within the AMF OTUs profiles in the resulting two nodes (main rule is shown above the node, and second rules are shown below the node). At each terminal node, the mean relative abundances of each AMF OTU are shown, together with the number of olive orchards for each group.

A multivariate regression tree was also calculated to summarize the relationships between AMF community composition derived from pyrosequencing analysis and environmental and agronomic variables with the most informative variables in each split shown in Figure 4. The tree explained >30% of the variability in AMF profiles, much of which were accounted by the first split based on exchangeable K and in a lower extend by altitude and sand and clay content (Figure 4). Then, climatic variables from sampled locations including total rainfall and evapotranspiration (ETP) followed by soil pH were the next best predictors for the second-order split. Two climatic variables (altitude and average rainfall), and nutrient contents of soil samples (including OM, C and extractable P and the C:N ratio) allowed differentiating five groups of soils that included three groups of soils showing high richness in OTUs and two groups of soils characterized by two specific OTUs each (Figure 4).

In our study we were able to identify some agronomic and environmental gradients driving the AMF community differentiation; however, we found some differences when using data derived from T-RFLP or pyrosequencing analysis. This could be due to the unequal sample size included in each data set (93 vs. 58 olive orchards, respectively). Another factor that is likely to have contributed to those differences is the usage of two different rRNA regions and methods, SSU for pyrosequencing and LSU for T-RFLP. These regions differ in their phylogenetic resolutions, and the methods of T-RFLP versus full amplicon sequencing differ as well in this respect as found in previous studies [49]. Consequently, in our study results from the pyrosequencing analysis should be interpreted with caution due to the smaller data set analysed and the possibility of introducing some bias due to the fact that from some olive orchards we did not amplified any *Glomeromycota* sequences. Nevertheless, we retrieved consistent results with both techniques. Thus, soil pH, textural characteristics, nutrient contents, and some climatic variables appear as the most important vectors driving the AMF community differentiation in olive. Soil texture has typically not been identified as being of great importance on AMF community composition until recently [43], [50]. Landis et al. [51] identified a texture effect in an oak savannah ecosystem, but the effects of texture could not be separated from plant community composition and soil N. A separate study [52] did find closely influences of clay, moisture, and pH in AMF composition in a maize agricultural system in Zimbabwe, along with strong effects of soil organic C and total N. On the other hand, Moebius-Clune et al. [51] studied AMF communities in an assemblage of maize fields across an eastern New York State landscape and found soil textural components as the most strongly related to AMF community differences followed by nutrient concentrations, particularly Mg, whereas soil P or pH, were less important.

These results demonstrate that when there are small differences in soil physicochemical characteristics, the composition of the AMF communities might be similar with an overlapping in the AMF assemblages among different agronomic or soil use practises [20]. All the data obtained in this work, reinforce the concept that the general AMF assemblage structure and composition in olive might be influenced primarily by soil type and climate and at less extent by host plant features (age, vegetative stages, host genotype) or agricultural practices as it has been shown in other woody crop such as vineyards [20], [53]. To our best knowledge this study provides the first evidence of a specific effect of such factors on AMF community composition in olive. Further research using a deeper pyrosequencing effort or more specific primers should be conducted to determine how this specific selection of AMF communities by the different olive varieties may be related to olive resilience to mycorrhization during the olive nursery process or to the successful establishment of those mycorrhized planting stocks when transplanted to soils in the different biogeographical areas (as identified by climatic and soil physicochemical properties) present in southern Spain.

## Supporting Information

Figure S1 Summary box-plots of Richness and Shannon diversity indexes derived from T-RFLP (93 olive orchards) and pyrosequencing analysis (43 olive orchards) grouped by the agronomic characteristics of the olive orchards sampled (Table S1).
(TIF)

Figure S2 Frequency of occurrence of the different *Glomeromycota* OTUs detected with primers NS31/AML2 and listed in Table 1 in 56 rhizosphere samples from 96 olive orchards sampled in Andalusia, southern Spain.
(TIF)

Table S1 Datasets, location and characteristics of the olive orchards sampled.
(PDF)

Table S2 Number of sequences and diversity indexes values obtained in the T-RFLP and pyrosequencing analysis in each orchard sampled.
(PDF)
