# Supplementary figures and images for: Arbuscular Mycorhizal Fungi Associated with the Olive Crop across the Andalusian Landscape: Factors Driving Community Differentiation
Source: PLoS One. 2014 May 5;9(5):e96397. doi: 10.1371/journal.pone.0096397 (PMC4010464; doi:10.1371/journal.pone.0096397)

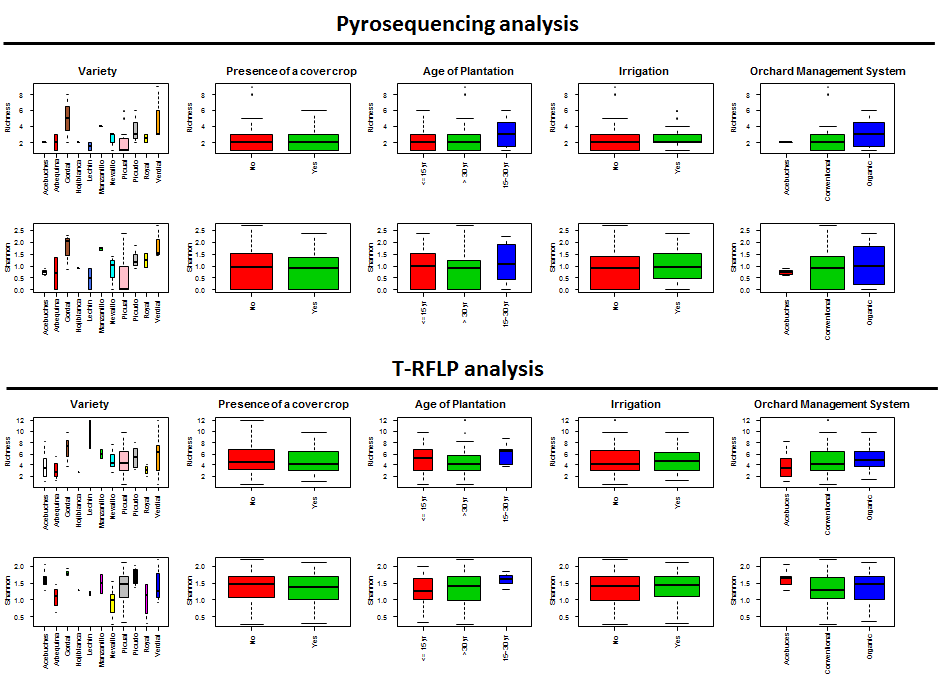

Supplement: Figure S1 — Summary box-plots of Richness and Shannon diversity indexes derived from T-RFLP (93 olive orchards) and pyrosequencing analysis (43 olive orchards) grouped by the agronomic characteristics of the olive orchards sampled (Table S1). (TIF) [file pone.0096397.s001.tif]

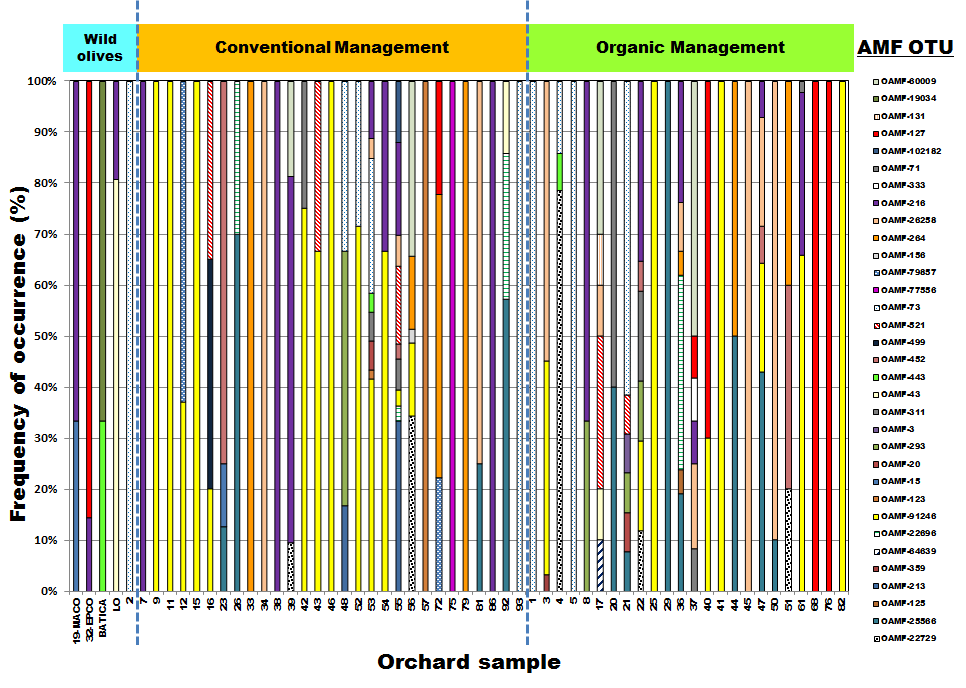

Supplement: Figure S2 — Frequency of occurrence of the different Glomeromycota OTUs detected with primers NS31/AML2 and listed in Table 1 in 56 rhizosphere samples from 96 olive orchards sampled in Andalusia, southern Spain. (TIF) [file pone.0096397.s002.tif]
