# Supplementary material for: Arbuscular Mycorhizal Fungi Associated with the Olive Crop across the Andalusian Landscape: Factors Driving Community Differentiation
Source: PLoS One. 2014 May 5;9(5):e96397. doi: 10.1371/journal.pone.0096397 (PMC4010464; doi:10.1371/journal.pone.0096397)
Supplement: Table S1 — Datasets, location and characteristics of the olive orchards sampled. (PDF) [file pone.0096397.s003.pdf]

Soil sample code	Geographic location			Agronomic characteristics					Soil physicochemical properties										Climatic characteristics						
	Latitude	Longitude	Orchard Management System	Province	Olive variety	Presence of a vegetative cover	Age of Plantation	Irrigation regimen	Cation Exchange Capacity	Soil Organic Matter (SOM; %)	C content (%)	N content (%)	C:N ratio	pH (KCl)	Extractable P (ppm)	Exchangeable K (ppm)	Clay content (%)	Sand content (%)	Total Rainfall	Average Rainfall	Evapotranspiration (mm)	Tmax (ºC)	Tmin (ºC)	Tmean (ºC)	Altitude (m)
**BAETICA**	36 ° 14 ´ 30.529 ´´N	5 ° 57 ´ 1.092 ´´W	Acebuches	Cádiz	Acebuches	Yes	>30 yr	No	32.6	8.6	5.0	0.4	13.8	7.1	ND	ND	21.7	68.6	1100.0	91.7	850.0	30.0	6.0	18.0	13
**LO**	36 ° 18 ´ 41.541 ´´N	5 ° 54 ´ 0.631 ´´W	Acebuches	Cádiz	Acebuches	Yes	>30 yr	No	20.2	2.6	1.5	0.2	10.2	5.0	ND	ND	17.8	74.4	700.0	58.3	950.0	30.0	6.0	18.0	52
**LOBA**	36 ° 18 ´ 24.528 ´´N	5 ° 52 ´ 55.508 ´´W	Acebuches	Cádiz	Acebuches	Yes	>30 yr	No	8.3	1.8	1.0	0.1	10.3	5.6	ND	ND	5.8	85.0	700.0	58.3	950.0	30.0	6.0	18.0	52
**S19-MACO**	37 ° 39 ´ 55.872 ´´N	4 ° 12 ´ 3.542 ´´W	Acebuches	Córdoba	Acebuches	Yes	>30 yr	No	19.6	4.4	2.6	0.2	12.1	6.4	10.6	280.0	14.0	56.5	630.5	72.1	895.8	23.1	10.9	17.5	269
**S31-LOMCO**	38 ° 26 ´ 21.258 ´´N	2 ° 46 ´ 1.864 ´´W	Acebuches	Córdoba	Acebuches	Yes	>30 yr	No	15.0	2.5	1.5	0.1	13.2	7.4	2.8	285.0	16.1	51.2	666.0	67.0	877.5	24.0	10.8	17.3	362
**S32-EPCO**	38 ° 26 ´ 23.918 ´´N	2 ° 46 ´ 1.236 ´´W	Acebuches	Córdoba	Acebuches	Yes	>30 yr	No	12.4	2.1	1.2	0.1	12.2	7.5	5.6	210.0	14.0	61.4	638.9	71.1	890.6	23.8	10.5	17.1	274
**S18**	37 ° 39 ´ 37.623 ´´N	4 ° 12 ´ 55.609 ´´W	Conventional	Córdoba	Arbequina	Yes	<= 15 yr	Yes	7.0	0.8	0.5	0.1	8.1	7.3	30.7	130.0	14.0	41.0	627.9	73.1	896.0	23.0	11.0	17.5	136
**S91**	37 ° 50 ´ 31.191 ´´N	5 ° 6 ´ 16.573 ´´W	Conventional	Córdoba	Arbequina	Yes	<= 15 yr	Yes	7.2	0.5	0.3	0.1	6.3	7.4	30.2	185.0	12.4	61.4	590.6	66.5	910.2	29.8	11.6	18.1	134
**S92**	37 ° 47 ´ 22.197 ´´N	5 ° 0 ´ 30.567 ´´W	Conventional	Córdoba	Arbequina	Yes	<= 15 yr	Yes	18.5	1.5	0.9	0.1	8.8	7.7	23.3	625.0	34.8	36.9	571.7	64.8	917.2	30.0	11.7	18.2	81
**S93**	37 ° 48 ´ 20.067 ´´N	4 ° 57 ´ 37.666 ´´W	Conventional	Córdoba	Arbequina	Yes	<= 15 yr	Yes	21.3	1.6	0.9	0.1	11.4	7.6	27.5	575.0	35.9	43.2	574.7	71.5	912.0	29.9	11.5	18.1	98
**S26**	37 ° 54 ´ 45.659 ´´N	4 ° 48 ´ 54.588 ´´W	Conventional	Córdoba	Hojiblanca	Yes	>30 yr	Yes	16.1	1.6	0.9	0.1	8.3	7.9	5.8	280.0	35.5	25.8	440.7	51.8	915.4	23.3	10.3	17.0	379
**S65**	38 ° 9 ´ 25.557 ´´N	4 ° 51 ´ 41.046 ´´W	Conventional	Córdoba	Nevadillo	No	>30 yr	No	14.4	3.4	1.9	0.2	12.1	5.1	6.2	210.0	6.4	58.5	584.3	63.0	829.7	24.1	10.1	15.5	566
**S67**	37 ° 38 ´ 19.563 ´´N	4 ° 29 ´ 7.363 ´´W	Conventional	Córdoba	Nevadillo	No	>30 yr	No	11.7	3.4	2.0	0.1	15.1	6.6	9.1	260.0	6.0	73.4	599.8	62.5	829.5	24.6	10.1	15.7	632
**S72**	37 ° 45 ´ 2.642 ´´N	3 ° 37 ´ 18.547 ´´W	Conventional	Córdoba	Nevadillo	No	>30 yr	No	19.2	1.6	0.9	0.1	11.3	5.2	6.4	38.0	4.8	55.7	617.7	62.9	870.8	24.5	10.3	15.5	326
**S69**	38 ° 5 ´ 1.861 ´´N	4 ° 41 ´ 44.308 ´´W	Conventional	Córdoba	Nevadillo	Yes	>30 yr	No	42.2	4.2	2.5	0.2	13.6	5.8	10.0	38.0	23.3	42.5	607.9	62.0	835.6	24.6	10.1	15.6	670
**S62**	38 ° 10 ´ 35.969 ´´N	4 ° 55 ´ 37.244 ´´W	Conventional	Córdoba	Nevadillo	Yes	>30 yr	Yes	17.4	2.8	1.6	0.2	10.8	5.9	9.8	190.0	13.5	52.8	607.4	60.8	829.9	25.2	10.6	16.5	589
**S11**	37 ° 53 ´ 28.541 ´´N	4 ° 51 ´ 49.169 ´´W	Conventional	Córdoba	Picual	No	>30 yr	No	22.0	1.0	0.6	0.1	8.6	7.7	6.6	710.0	41.9	23.7	529.5	60.2	872.2	21.5	10.6	16.9	249
**S2**	37 ° 40 ´ 51.568 ´´N	4 ° 25 ´ 31.668 ´´W	Conventional	Córdoba	Picual	No	<= 15 yr	No	23.7	1.1	0.6	0.1	10.6	7.6	5.1	555.0	51.7	17.3	547.9	64.8	877.9	21.2	10.4	16.2	188
**S23**	37 ° 38 ´ 10.801 ´´N	4 ° 29 ´ 7.515 ´´W	Conventional	Córdoba	Picual	No	15-30 yr	No	27.6	0.9	0.6	0.1	9.1	7.5	12.5	570.0	56.6	13.4	458.8	55.1	909.2	21.8	10.4	16.4	371
**S30**	38 ° 26 ´ 23.822 ´´N	2 ° 46 ´ 2.227 ´´W	Conventional	Córdoba	Picual	No	<= 15 yr	Yes	16.3	0.9	0.5	0.1	8.2	7.8	12.8	280.0	46.5	13.0	453.9	53.1	913.1	24.3	10.7	17.5	408
**S7**	37 ° 49 ´ 12.034 ´´N	3 ° 6 ´ 2.677 ´´W	Conventional	Córdoba	Picual	Yes	15-30 yr	No	13.7	1.1	0.7	0.1	9.4	7.8	3.4	128.0	39.8	20.6	542.6	60.6	863.6	24.7	10.1	15.7	421
**S59**	38 ° 7 ´ 16.381 ´´N	4 ° 51 ´ 34.725 ´´W	Conventional	Córdoba	Picual	Yes	>30 yr	Yes	24.4	1.4	0.8	0.1	9.0	7.5	5.4	390.0	47.0	15.3	473.5	46.8	875.7	27.1	11.7	18.2	553
**S60**	37 ° 45 ´ 9.285 ´´N	3 ° 37 ´ 7.773 ´´W	Conventional	Córdoba	Picual	Yes	>30 yr	Yes	12.2	2.1	1.2	0.1	10.8	7.8	19.2	580.0	32.3	30.6	495.1	47.2	868.5	27.3	11.7	18.2	523
**S6**	37 ° 47 ´ 57.833 ´´N	3 ° 2 ´ 13.017 ´´W	Conventional	Córdoba	Picudo	No	>30 yr	No	26.7	2.0	1.1	0.1	9.5	7.6	2.7	230.0	48.9	15.5	542.6	60.6	863.6	27.2	11.8	18.3	412
**S9**	37 ° 52 ´ 24.991 ´´N	4 ° 52 ´ 31.767 ´´W	Conventional	Córdoba	Picudo	No	>30 yr	No	24.1	1.7	1.0	0.1	8.9	7.6	4.9	270.0	44.5	30.8	543.4	60.7	865.8	27.7	11.4	17.0	391
**S90**	37 ° 17 ´ 41.759 ´´N	3 ° 18 ´ 51.233 ´´W	Conventional	Granada	Picual	No	>30 yr	Yes	12.6	1.8	1.1	0.1	11.7	7.7	10.7	325.0	19.6	56.2	582.1	71.9	769.8	25.7	8.4	14.5	1018
**S33**	38 ° 26 ´ 31.952 ´´N	2 ° 46 ´ 11.894 ´´W	Conventional	Jaén	Picual	No	>30 yr	No	22.6	2.3	1.3	0.1	10.1	7.6	3.6	360.0	31.9	25.8	701.4	75.4	712.5	24.0	10.8	17.3	1047
**S34**	37 ° 38 ´ 10.139 ´´N	4 ° 29 ´ 5.952 ´´W	Conventional	Jaén	Picual	No	>30 yr	No	26.5	0.9	0.5	0.1	8.8	7.6	10.6	530.0	52.1	12.1	509.4	73.8	740.3	24.3	10.9	17.4	508
**S42**	37 ° 20 ´ 27.258 ´´N	5 ° 15 ´ 53.537 ´´W	Conventional	Jaén	Picual	No	>30 yr	No	23.7	3.2	1.8	0.2	11.5	7.5	5.4	690.0	35.3	41.9	653.3	70.5	836.7	22.7	8.1	14.8	808
**S75**	37 ° 47 ´ 37.979 ´´N	3 ° 26 ´ 44.586 ´´W	Conventional	Jaén	Picual	No	>30 yr	No	22.3	2.5	1.4	0.1	10.2	7.6	4.7	420.0	44.6	23.7	583.9	73.5	845.9	23.5	9.1	14.3	982
**S81**	37 ° 33 ´ 36.793 ´´N	4 ° 1 ´ 50.570 ´´W	Conventional	Jaén	Picual	No	>30 yr	No	13.2	2.3	1.3	0.2	8.3	7.8	7.5	465.0	30.2	35.7	548.4	48.6	829.6	26.3	10.7	16.3	638
**S86**	37 ° 33 ´ 20.733 ´´N	4 ° 7 ´ 0.781 ´´W	Conventional	Jaén	Picual	No	15-30 yr	No	15.7	1.5	0.9	0.1	9.8	7.6	4.0	410.0	38.4	30.5	548.4	48.6	829.6	27.0	11.1	16.6	581
**S88**	37 ° 18 ´ 3.106 ´´N	3 ° 18 ´ 15.740 ´´W	Conventional	Jaén	Picual	No	>30 yr	No	11.1	1.4	0.8	0.1	9.8	7.9	8.2	260.0	24.7	33.3	501.6	50.5	854.4	25.4	8.4	14.4	470
**S46**	37 ° 20 ´ 45.233 ´´N	5 ° 16 ´ 37.354 ´´W	Conventional	Jaén	Picual	No	<= 15 yr	Yes	20.0	1.7	1.0	0.1	9.7	7.7	5.8	900.0	39.7	24.6	634.5	72.8	842.9	22.6	7.8	14.5	765
**S77**	37 ° 40 ´ 36.307 ´´N	3 ° 56 ´ 26.528 ´´W	Conventional	Jaén	Picual	No	>30 yr	Yes	19.0	1.2	0.7	0.1	7.6	7.5	5.3	265.0	43.1	20.4	583.9	73.5	845.9	24.0	9.3	14.5	704
**S84**	37 ° 32 ´ 47.176 ´´N	4 ° 0 ´ 39.959 ´´W	Conventional	Jaén	Picual	No	>30 yr	Yes	12.2	2.1	1.2	0.1	10.2	7.9	9.7	380.0	34.6	28.2	529.1	46.0	830.2	26.8	11.0	16.6	574
**S35**	38 ° 26 ´ 30.561 ´´N	2 ° 46 ´ 14.167 ´´W	Conventional	Jaén	Picual	Yes	15-30 yr	No	30.4	1.5	0.8	0.1	10.5	7.6	8.8	350.0	56.0	13.7	571.4	73.4	816.3	24.0	10.1	16.8	942
**S38**	38 ° 25 ´ 35.736 ´´N	2 ° 44 ´ 12.183 ´´W	Conventional	Jaén	Picual	Yes	>30 yr	No	7.2	1.0	0.6	0.1	8.5	6.9	10.8	380.0	17.9	60.0	630.3	70.9	836.7	24.3	10.4	17.0	737
**S43**	37 ° 20 ´ 26.495 ´´N	5 ° 15 ´ 54.408 ´´W	Conventional	Jaén	Picual	Yes	>30 yr	No	18.5	2.8	1.6	0.2	10.0	7.5	14.1	790.0	29.7	44.3	643.6	69.9	844.1	22.9	8.2	14.9	843
**S79**	37 ° 33 ´ 38.711 ´´N	4 ° 1 ´ 27.772 ´´W	Conventional	Jaén	Picual	Yes	<= 15 yr	No	8.4	1.6	1.0	0.1	9.5	5.3	14.6	230.0	13.7	59.6	604.9	71.1	867.9	25.3	10.9	16.0	954
**S15**	37 ° 42 ´ 34.754 ´´N	4 ° 18 ´ 4.628 ´´W	Conventional	Jaén	Picual	Yes	>30 yr	Yes	21.1	1.8	1.1	0.1	10.5	7.8	43.1	630.0	56.6	15.6	609.2	72.0	907.9	21.8	10.7	16.9	708

Soil sample code	Geographic location			Agronomic characteristics					Soil physicochemical properties										Climatic characteristics						
	Latitude	Longitude	Orchard Management System	Province	Olive variety	Presence of a vegetative cover	Age of Plantation	Irrigation regimen	Cation Exchange Capacity	Soil Organic Matter (SOM; %)	C content (%)	N content (%)	C:N ratio	pH (KCl)	Extractable P (ppm)	Exchangeable K (ppm)	Clay content (%)	Sand content (%)	Total Rainfall	Average Rainfall	Evapotranspiration (mm)	Tmax (ºC)	Tmin (ºC)	Tmean (ºC)	Altitude (m)
**S39**	38 ° 25 ′ 37.262 ′′N	2 ° 44 ′ 12.755 ′′W	Conventional	Jaén	Picual	Yes	<= 15 yr	Yes	8.5	1.0	0.6	0.1	7.5	4.5	9.1	320.0	20.8	52.2	630.3	70.9	836.7	21.2	9.0	14.6	742
**S14**	37 ° 42 ′ 35.065 ′′N	4 ° 18 ′ 5.859 ′′W	Conventional	Jaén	Royal	No	>30 yr	No	19.4	3.2	1.8	0.2	12.3	7.8	6.2	560.0	33.6	29.5	609.2	72.0	907.9	21.8	10.7	16.9	893
**S12**	37 ° 46 ′ 6.732 ′′N	4 ° 39 ′ 38.977 ′′W	Conventional	Jaén	Royal	Yes	>30 yr	Yes	23.5	1.4	0.8	0.1	8.0	7.6	27.5	680.0	45.0	20.6	609.2	72.0	907.9	21.6	10.6	16.8	683
**S13**	37 ° 42 ′ 33.743 ′′N	4 ° 18 ′ 8.000 ′′W	Conventional	Jaén	Royal	Yes	>30 yr	Yes	21.1	1.4	0.8	0.1	7.3	7.7	20.2	705.0	57.2	12.1	609.2	72.0	907.9	21.7	10.7	16.9	737
**S16**	37 ° 42 ′ 32.434 ′′N	4 ° 18 ′ 3.117 ′′W	Conventional	Jaén	Royal	Yes	>30 yr	Yes	21.7	1.5	0.9	0.1	7.9	7.7	16.0	950.0	48.0	14.7	609.2	72.0	907.9	22.0	10.5	16.9	628
**S52**	37 ° 17 ′ 49.307 ′′N	5 ° 23 ′ 6.999 ′′W	Conventional	Sevilla	Gordal	No	>30 yr	No	22.2	2.1	1.2	0.1	9.2	7.5	13.4	380.0	44.2	17.9	532.2	55.3	918.6	26.4	11.5	18.0	154
**S53**	37 ° 18 ′ 20.070 ′′N	5 ° 23 ′ 32.298 ′′W	Conventional	Sevilla	Gordal	No	>30 yr	No	22.6	2.3	1.3	0.1	9.4	7.5	14.9	385.0	46.9	18.3	532.2	55.3	918.6	26.5	11.5	18.1	155
**S54**	37 ° 25 ′ 18.281 ′′N	5 ° 50 ′ 58.411 ′′W	Conventional	Sevilla	Lechin	No	>30 yr	No	2.8	0.3	0.2	0.0	6.4	6.3	4.0	45.0	3.3	91.6	537.2	57.1	941.4	26.4	11.4	17.9	150
**S57**	37 ° 36 ′ 15.413 ′′N	4 ° 7 ′ 8.734 ′′W	Conventional	Sevilla	Lechin	No	>30 yr	No	15.9	1.3	0.8	0.1	8.6	7.5	13.3	390.0	21.6	61.0	542.3	56.5	929.2	26.8	11.4	18.0	151
**S56**	37 ° 38 ′ 25.782 ′′N	4 ° 29 ′ 5.570 ′′W	Conventional	Sevilla	Manzanillo	No	15-30 yr	No	13.0	0.9	0.5	0.1	8.2	7.3	9.0	125.0	22.0	71.4	541.3	56.7	932.4	26.8	11.4	18.1	156
**S48**	37 ° 21 ′ 58.160 ′′N	5 ° 23 ′ 45.887 ′′W	Conventional	Sevilla	Verdial	No	>30 yr	No	24.4	4.6	2.7	0.2	11.5	7.5	4.2	475.0	28.6	42.2	531.4	55.3	939.5	22.8	7.8	14.5	139
**S55**	37 ° 37 ′ 35.704 ′′N	4 ° 4 ′ 15.422 ′′W	Conventional	Sevilla	Verdial	No	>30 yr	No	28.0	1.9	1.1	0.1	8.3	7.4	4.8	260.0	51.0	17.0	539.4	56.8	935.7	26.6	11.6	18.1	121
**S49**	37 ° 21 ′ 58.160 ′′N	5 ° 23 ′ 45.887 ′′W	Conventional	Sevilla	Verdial	No	<= 15 yr	Yes	17.4	2.1	1.2	0.1	8.7	7.7	23.3	590.0	26.9	40.8	527.2	55.2	939.4	22.9	7.8	14.5	136
**S58**	38 ° 8 ′ 0.486 ′′N	3 ° 53 ′ 19.563 ′′W	Conventional	Sevilla	Verdial	No	<= 15 yr	Yes	5.9	0.7	0.4	0.1	8.6	6.0	52.4	290.0	7.1	91.4	537.2	67.1	948.6	27.0	11.9	18.3	148
**S17**	37 ° 42 ′ 13.476 ′′N	4 ° 18 ′ 9.932 ′′W	Organic	Córdoba	Manzanillo	Yes	<= 15 yr	Yes	8.3	1.6	1.0	0.1	9.5	7.0	41.6	310.0	13.1	39.6	627.9	73.1	896.0	22.1	10.5	16.9	140
**S66**	38 ° 9 ′ 23.563 ′′N	4 ° 48 ′ 47.867 ′′W	Organic	Córdoba	Nevadillo	No	>30 yr	No	12.6	2.9	1.7	0.1	14.1	5.8	13.8	114.0	6.0	75.5	599.8	62.5	829.5	24.3	10.3	15.7	623
**S71**	37 ° 45 ′ 9.285 ′′N	3 ° 37 ′ 7.773 ′′W	Organic	Córdoba	Nevadillo	No	>30 yr	No	11.1	3.8	2.2	0.2	14.7	7.1	6.4	190.0	6.4	67.9	617.7	62.9	870.8	24.4	10.2	15.5	393
**S61**	38 ° 10 ′ 35.969 ′′N	4 ° 55 ′ 37.244 ′′W	Organic	Córdoba	Nevadillo	Yes	>30 yr	No	13.5	5.3	3.1	0.2	14.6	7.1	12.2	535.0	9.3	67.9	522.0	63.1	985.9	27.4	11.8	18.3	537
**S63**	38 ° 9 ′ 58.501 ′′N	4 ° 54 ′ 13.709 ′′W	Organic	Córdoba	Nevadillo	Yes	>30 yr	No	21.5	2.8	1.7	0.1	12.7	6.7	10.5	128.0	9.3	45.0	492.0	77.3	826.6	25.4	11.0	16.8	546
**S64**	38 ° 9 ′ 58.501 ′′N	4 ° 54 ′ 13.709 ′′W	Organic	Córdoba	Nevadillo	Yes	>30 yr	No	20.7	3.5	2.0	0.2	13.3	5.8	8.2	129.0	8.0	60.8	584.3	63.0	829.7	24.3	10.3	15.8	565
**S68**	38 ° 9 ′ 23.563 ′′N	4 ° 48 ′ 47.867 ′′W	Organic	Córdoba	Nevadillo	Yes	>30 yr	No	20.9	3.8	2.2	0.1	15.7	5.9	5.6	106.0	8.2	73.4	603.1	62.0	830.8	24.8	10.1	15.7	481
**S70**	38 ° 5 ′ 2.202 ′′N	4 ° 41 ′ 45.342 ′′W	Organic	Córdoba	Nevadillo	Yes	>30 yr	No	40.0	7.8	4.5	0.3	16.1	6.3	9.5	240.0	20.7	46.3	607.9	62.0	835.6	25.2	10.7	16.1	665
**S1**	37 ° 46 ′ 12.107 ′′N	4 ° 39 ′ 39.711 ′′W	Organic	Córdoba	Picual	No	<= 15 yr	No	25.2	1.5	0.9	0.1	12.3	7.6	5.3	385.0	50.4	18.0	548.9	66.1	875.1	22.4	10.8	17.5	171
**S28**	37 ° 20 ′ 24.575 ′′N	3 ° 53 ′ 20.997 ′′W	Organic	Córdoba	Picual	No	>30 yr	No	15.9	1.9	1.1	0.1	9.0	7.9	6.2	350.0	37.5	19.7	461.0	54.6	910.7	23.9	10.5	17.3	314
**S10**	37 ° 52 ′ 24.710 ′′N	4 ° 52 ′ 33.111 ′′W	Organic	Córdoba	Picual	No	>30 yr	Yes	27.6	1.5	0.9	0.1	9.7	7.7	4.4	830.0	48.4	20.6	529.5	60.2	872.2	22.4	10.7	17.4	246
**S20**	37 ° 38 ′ 39.051 ′′N	4 ° 13 ′ 24.604 ′′W	Organic	Córdoba	Picual	No	<= 15 yr	Yes	43.5	1.5	0.9	0.1	12.5	7.6	3.2	760.0	78.1	8.7	458.8	55.1	909.2	20.9	9.8	15.7	367
**S24**	37 ° 41 ′ 31.298 ′′N	4 ° 12 ′ 12.675 ′′W	Organic	Córdoba	Picual	No	15-30 yr	Yes	23.0	1.2	0.7	0.1	8.9	7.6	4.0	360.0	50.6	13.4	464.9	55.7	908.8	23.7	10.9	17.6	354
**S22**	37 ° 41 ′ 33.147 ′′N	4 ° 12 ′ 12.786 ′′W	Organic	Córdoba	Picual	Yes	<= 15 yr	Yes	23.3	1.2	0.7	0.1	9.6	7.7	3.4	450.0	49.3	18.2	458.8	55.1	909.2	21.1	9.9	15.7	373
**S25**	37 ° 55 ′ 59.724 ′′N	4 ° 48 ′ 22.986 ′′W	Organic	Córdoba	Picual	Yes	<= 15 yr	Yes	19.6	1.1	0.6	0.1	7.6	7.7	8.3	360.0	31.8	20.7	440.7	51.8	915.4	23.8	10.9	17.6	347
**S29**	37 ° 55 ′ 33.414 ′′N	3 ° 6 ′ 32.115 ′′W	Organic	Córdoba	Picual	Yes	<= 15 yr	Yes	17.8	1.1	0.6	0.1	8.0	7.7	6.4	300.0	54.5	10.4	453.9	53.1	913.1	23.9	10.5	17.3	403
**S4**	37 ° 50 ′ 11.732 ′′N	3 ° 3 ′ 48.816 ′′W	Organic	Córdoba	Picual	Yes	>30 yr	Yes	28.3	2.3	1.3	0.2	8.8	7.6	22.9	435.0	35.6	23.1	542.6	60.6	863.6	23.9	10.1	16.6	402
**S5**	37 ° 50 ′ 5.148 ′′N	3 ° 6 ′ 3.854 ′′W	Organic	Córdoba	Picual	Yes	15-30 yr	Yes	19.6	1.5	0.9	0.1	9.7	7.7	4.9	180.0	46.1	20.6	542.6	60.6	863.6	23.2	8.0	14.7	411
**S27**	37 ° 51 ′ 13.986 ′′N	3 ° 1 ′ 19.468 ′′W	Organic	Córdoba	Picudo	No	>30 yr	No	14.3	2.1	1.2	0.1	8.5	7.8	8.4	300.0	25.5	35.7	471.1	54.8	907.8	23.8	10.5	17.3	357
**S8**	37 ° 50 ′ 53.245 ′′N	3 ° 5 ′ 51.031 ′′W	Organic	Córdoba	Picudo	Yes	>30 yr	No	27.0	1.8	1.1	0.1	8.8	7.6	3.0	335.0	47.6	20.6	543.4	60.7	865.8	24.6	8.4	14.3	396
**S21**	37 ° 39 ′ 2.932 ′′N	4 ° 13 ′ 30.750 ′′W	Organic	Córdoba	Picudo	Yes	<= 15 yr	Yes	18.9	1.1	0.6	0.1	8.0	7.7	3.4	420.0	40.0	30.9	458.8	55.1	909.2	20.3	10.0	15.4	372
**S3**	37 ° 41 ′ 10.736 ′′N	3 ° 55 ′ 16.250 ′′W	Organic	Córdoba	Picudo	Yes	<= 15 yr	Yes	14.1	1.5	0.9	0.1	8.0	7.9	52.4	375.0	34.9	28.3	542.6	60.6	863.6	24.1	10.6	17.4	408
**S89**	37 ° 38 ′ 44.508 ′′N	4 ° 30 ′ 11.757 ′′W	Organic	Granada	Picual	Yes	>30 yr	No	14.3	2.6	1.5	0.2	9.6	7.7	6.2	310.0	24.0	22.4	582.1	71.9	769.8	27.7	11.4	17.1	967
**S74**	37 ° 47 ′ 38.368 ′′N	3 ° 26 ′ 44.711 ′′W	Organic	Jaén	Picual	No	>30 yr	No	19.0	2.3	1.4	0.1	9.7	7.7	8.2	495.0	36.9	28.1	492.0	77.3	826.6	26.2	10.8	16.6	1007
**S37**	38 ° 23 ′ 50.427 ′′N	2 ° 44 ′ 57.377 ′′W	Organic	Jaén	Picual	No	15-30 yr	Yes	10.9	1.4	0.8	0.1	10.2	7.4	4.3	480.0	24.6	52.2	630.3	70.9	836.7	23.6	10.1	16.5	738
**S41**	38 ° 26 ′ 15.772 ′′N	2 ° 43 ′ 58.343 ′′W	Organic	Jaén	Picual	No	>30 yr	Yes	20.4	3.0	1.7	0.1	12.3	7.6	2.5	625.0	32.3	48.7	653.3	70.5	836.7	22.7	8.1	14.8	812
**S45**	37 ° 38 ′ 24.949 ′′N	4 ° 29 ′ 7.307 ′′W	Organic	Jaén	Picual	No	<= 15 yr	Yes	12.8	1.7	1.0	0.1	9.1	7.7	5.0	310.0	24.2	24.3	654.2	72.1	845.2	23.0	8.1	14.8	765

Soil sample code	Geographic location			Agronomic characteristics					Soil physicochemical properties										Climatic characteristics						
	Latitude	Longitude	Orchard Management System	Province	Olive variety	Presence of a vegetative cover	Age of Plantation	Irrigation regimen	Cation Exchange Capacity	Soil Organic Matter (SOM; %)	C content (%)	N content (%)	C:N ratio	pH (KCl)	Extractable P (ppm)	Exchangeable K (ppm)	Clay content (%)	Sand content (%)	Total Rainfall	Average Rainfall	Evapotranspiration (mm)	Tmax (ºC)	Tmin (ºC)	Tmean (ºC)	Altitude (m)
**S73**	37 ° 47 ´ 38.368 ´´N	3 ° 26 ´ 44.711 ´´W	Organic	Jaén	Picual	Yes	>30 yr	No	21.9	4.4	2.6	0.3	9.9	7.5	59.8	890.0	30.7	38.3	492.0	77.3	826.6	26.0	10.7	16.5	1063
**S83**	37 ° 32 ´ 56.517 ´´N	4 ° 2 ´ 17.564 ´´W	Organic	Jaén	Picual	Yes	>30 yr	No	14.4	3.2	1.9	0.2	11.7	7.8	6.9	540.0	29.8	35.9	548.4	48.6	829.6	26.5	10.8	16.3	582
**S85**	37 ° 32 ´ 48.041 ´´N	4 ° 0 ´ 37.444 ´´W	Organic	Jaén	Picual	Yes	>30 yr	No	17.0	3.7	2.2	0.2	13.5	7.7	5.0	640.0	42.5	28.2	548.4	48.6	829.6	26.9	11.0	16.6	588
**S36**	38 ° 23 ´ 49.713 ´´N	2 ° 44 ´ 57.091 ´´W	Organic	Jaén	Picual	Yes	15-30 yr	Yes	9.8	1.6	0.9	0.1	10.3	7.3	5.6	500.0	20.4	53.8	630.3	70.9	836.7	23.9	10.0	16.7	741
**S40**	38 ° 26 ´ 13.654 ´´N	2 ° 44 ´ 8.952 ´´W	Organic	Jaén	Picual	Yes	15-30 yr	Yes	8.5	1.7	1.0	0.1	11.2	6.7	5.5	385.0	12.0	60.0	630.3	70.9	836.7	22.5	8.5	15.0	741
**S44**	37 ° 20 ´ 49.089 ´´N	5 ° 16 ´ 5.531 ´´W	Organic	Jaén	Picual	Yes	>30 yr	Yes	20.0	2.5	1.5	0.2	9.7	7.6	5.6	870.0	38.1	39.3	649.1	71.9	843.3	23.0	8.2	14.9	838
**S76**	37 ° 40 ´ 38.840 ´´N	3 ° 56 ´ 26.233 ´´W	Organic	Jaén	Picual	Yes	>30 yr	Yes	26.3	2.3	1.3	0.2	8.3	7.5	4.5	375.0	38.4	37.3	583.9	73.5	845.9	23.8	9.2	14.4	708
**S78**	37 ° 38 ´ 45.700 ´´N	4 ° 30 ´ 9.904 ´´W	Organic	Jaén	Picual	Yes	<= 15 yr	Yes	11.3	2.9	1.7	0.2	9.2	7.5	68.5	660.0	18.7	43.0	604.9	71.1	867.9	25.3	11.0	16.0	951
**S80**	37 ° 33 ´ 38.711 ´´N	4 ° 1 ´ 27.772 ´´W	Organic	Jaén	Picual	Yes	>30 yr	Yes	13.2	2.6	1.5	0.1	10.8	7.8	4.7	555.0	26.7	37.8	548.4	48.6	829.6	24.7	8.4	14.3	637
**S82**	37 ° 32 ´ 53.592 ´´N	4 ° 1 ´ 55.925 ´´W	Organic	Jaén	Picual	Yes	>30 yr	Yes	24.5	3.6	2.1	0.2	12.9	7.5	4.9	940.0	48.1	33.7	548.4	48.6	829.6	26.4	10.7	16.3	627
**S87**	37 ° 33 ´ 21.742 ´´N	4 ° 7 ´ 0.430 ´´W	Organic	Jaén	Picual	Yes	>30 yr	Yes	13.0	2.9	1.7	0.1	12.0	8.0	6.9	370.0	34.3	30.8	501.6	50.5	854.4	27.1	11.1	16.7	471
**S47**	37 ° 20 ´ 45.998 ´´N	5 ° 16 ´ 38.027 ´´W	Organic	Sevilla	Gordal	Yes	>30 yr	No	23.7	3.2	1.8	0.2	10.9	7.6	6.3	520.0	31.2	39.7	527.2	55.2	939.4	22.7	7.8	14.5	139
**S50**	37 ° 16 ´ 17.631 ´´N	5 ° 21 ´ 58.910 ´´W	Organic	Sevilla	Picual	No	<= 15 yr	Yes	17.8	2.4	1.4	0.1	9.9	7.8	5.2	345.0	27.7	43.4	531.4	55.3	939.5	23.3	8.0	14.7	139
**S51**	37 ° 18 ´ 2.646 ´´N	5 ° 22 ´ 11.866 ´´W	Organic	Sevilla	Verdial	No	<= 15 yr	Yes	14.8	2.0	1.2	0.1	9.6	7.9	6.2	250.0	24.7	45.6	531.4	55.3	939.5	26.3	11.5	18.0	139
