# Supplementary material for: Arbuscular Mycorhizal Fungi Associated with the Olive Crop across the Andalusian Landscape: Factors Driving Community Differentiation
Source: PLoS One. 2014 May 5;9(5):e96397. doi: 10.1371/journal.pone.0096397 (PMC4010464; doi:10.1371/journal.pone.0096397)
Supplement: Table S2 — Number of sequences and diversity indexes values obtained in the T-RFLP and pyrosequencing analysis in each orchard sampled. (PDF) [file pone.0096397.s004.pdf]

Soil sample code	T-RFLP analysis		Pyrosequencing analysis (Number of sequences)			Pyrosequencing analysis (alpha-diversity)	
	Richness	Shannon	Total	Fungal	Glomeromycota	Richness*	Shannon
**S1**	2	0.99	116	14	11	1	0.00
**S2**	0.5	0.35	113	2	1	1*	---
**S3**	5.5	1.91	196	35	31	3	1.16
**S4**	3.75	1.78	188	14	14	3	0.95
**S5**	3.75	1.44	173	21	14	1	0.00
**S6**	3.5	1.36	330	0	0	---	---
**S7**	8.75	1.31	86	36	36	1	0.00
**S8**	7	1.79	112	16	6	2	0.92
**S9**	5.25	2.01	59	3	1	1*	0.00
**S10**	3.75	1.43	132	0	0	---	---
**S11**	5	1.58	108	3	1	1*	---
**S12**	3.5	1.47	137	29	27	2	0.95
**S13**	2	0.30	110	2	0	---	---
**S14**	4	1.41	103	0	0	---	---
**S15**	3	1.18	302	3	1	1*	---
**S16**	2.75	0.89	275	20	20	3	1.51
**S17**	5.25	1.19	123	10	10	4	1.79
**S18**	1.25	1.05	64	1	0	---	---
**S19-MACO**	5.25	1.69	117	9	3	2*	---
**S20**	5	1.59	60	8	5	2	0.97
**S21**	8	1.52	100	13	13	6	1.85
**S22**	9.75	1.97	80	19	17	6	2.38
**S23**	7	1.83	92	8	8	3	1.06
**S24**	4	1.72	62	3	0	---	---
**S25**	6.5	1.88	50	14	7	1	0.00
**S26**	2.75	1.28	84	10	10	2	0.88
**S27**	3.25	1.48	60	1	0	---	---
**S28**	2.75	1.50	68	0	0	---	---
**S29**	1.5	0.99	131	3	2	1*	---
**S30**	3.5	0.79	78	1	0	---	---
**S31-LOMCO**	8.25	1.58	200	3	0	---	---
**S32-EPCO**	3.75	1.70	87	10	7	2	0.59
**S33**	2.5	1.04	105	8	5	1	0.00
**S34**	4.25	0.98	71	20	9	1	0.00
**S35**	6.5	1.64	695	5	0	---	---

Soil sample code	T-RFLP analysis		Pyrosequencing analysis (Number of sequences)			Pyrosequencing analysis (alpha-diversity)	
	Richness	Shannon	Total	Fungal	Glomeromycota	Richness	Shannon
**S36**	6.75	1.57	90	28	21	6	2.22
**S37**	6.25	1.49	108	12	12	5	2.25
**S38**	5	1.74	49	17	6	1	0.00
**S39**	9.5	1.30	99	32	32	2	0.52
**S40**	5	1.50	112	20	10	2	0.88
**S41**	4.75	1.45	74	5	2	1*	---
**S42**	7.5	1.88	191	6	4	2*	---
**S43**	6.5	2.04	81	9	3	2*	---
**S44**	4.75	1.94	114	5	2	2*	---
**S45**	7.75	1.69	113	4	2	1*	---
**S46**	5.25	1.93	88	38	1	1*	---
**S47**	3.75	1.73	218	23	14	5	2.02
**S48**	0.5	1.07	129	19	6	3	1.46
**S49**	3	0.93	72	6	0	---	---
**S50**	6.25	1.25	152	14	10	2	0.47
**S51**	7.5	1.79	65	6	5	3	1.52
**S52**	7.25	1.77	143	12	7	2	0.86
**S53**	9.75	1.93	107	60	53	8	2.32
**S54**	12	1.26	98	7	3	2*	---
**S55**	12	2.19	97	39	33	9	2.71
**S56**	6.75	1.77	130	35	35	4	1.64
**S57**	7	1.14	186	3	1	1*	---
**S58**	6.25	1.25	166	7	0	---	---
**S59**	5.75	1.42	257	0	0	---	---
**S60**	4.25	1.96	88	2	0	---	---
**S61**	4	1.23	252	52	44	3	1.05
**S62**	4.5	0.60	263	2	0	---	---
**S63**	6.25	1.55	374	3	0	---	---
**S64**	6.5	1.00	13	0	0	---	---
**S65**	3.75	0.99	52	1	0	---	---
**S66**	7.75	0.98	70	21	0	---	---
**S67**	4	1.18	244	23	0	---	---
**S68**	4.75	0.92	29	7	6	1	0.00
**S69**	4	0.65	281	11	0	---	---
**S70**	3.75	0.36	102	6	0	---	---

Soil sample code	T-RFLP analysis		Pyrosequencing analysis (Number of sequences)			Pyrosequencing analysis (alpha-diversity)	
	Richness	Shannon	Total	Fungal	Glomeromycota	Richness	Shannon
**S71**	5.5	1.14	395	11	0	---	---
**S72**	2.75	0.30	97	9	9	3	1.44
**S73**	1.75	0.72	25	3	0	---	---
**S74**	2.75	1.67	145	2	0	---	---
**S75**	7.25	1.69	146	35	5	1	0.00
**S76**	7.25	2.12	64	5	1	1*	---
**S77**	3.75	1.73	229	10	0	---	---
**S78**	7	1.12	181	11	0	---	---
**S79**	3.75	1.29	119	3	1	1*	---
**S80**	3	1.46	207	11	0	---	---
**S81**	2.75	0.37	125	4	4	---	---
**S82**	2	0.96	30	7	6	1	0.00
**S83**	4	0.98	0	0	0	---	---
**S84**	3.25	1.07	556	2	0	---	---
**S85**	2.25	1.07	262	10	0	---	---
**S86**	3.75	1.65	126	4	4	---	---
**S87**	2.5	0.78	154	3	0	---	---
**S88**	3.25	1.64	63	0	0	---	---
**S89**	4.5	0.91	114	11	0	---	---
**S90**	6	1.24	129	11	0	---	---
**S91**	5.5	1.16	116	5	0	---	---
**S92**	3	1.46	145	12	7	3	1.38
**S93**	2.5	0.63	241	5	5	1	0.00
**BAETICA**	2	1.44	177	24	24	2	0.92
**LO**	3	1.29	352	31	26	2	0.71
**LOBA**	1	2.04	0	0	0	---	---

*** Samples with <5 Glomeromycota sequences were not included in the statistical analysis and in Figure S1**
